# Influence of Carbonation on the Properties of Steel Slag–Magnesium Silicate Hydrate (MSH) Cement

**DOI:** 10.3390/ma16206737

**Published:** 2023-10-18

**Authors:** Tian Zeng, Zhiqi Hu, Chengran Huang, Jun Chang

**Affiliations:** 1 Faculty of Infrastructure Engineering, Dalian University of Technology, Dalian 116024, China; tianzeng_dut@163.com (T.Z.); anshanhzq@126.com (Z.H.); 2 Institute of Materials and Metallurgy, University of Science and Technology Liaoning, Anshan 114031, China; huang15566286695@163.com

**Keywords:** magnesium silicate hydrate cement, carbonation, steel slag, drying shrinkage property

## Abstract

Magnesium silicate hydrate (MSH) cement has the advantages of low energy consumption, minimal environmental pollution, carbon negativity, and reduced alkalinity, but excessive drying shrinkage inhibits its application. This paper analyzed the influence of steel slag (SS) dosage, carbon dioxide partial pressure, and carbonation curing time on the compressive strength, shrinkage rate, and phase composition of MSH cement. Various analysis methods, including X-ray diffraction (XRD), thermogravimetric analysis (TGA), scanning electron microscopy (SEM), and mercury intrusion porosimetry (MIP), were used to study the hydration products and microstructure. The results showed that under normal curing conditions, MSH cement mixed with different steel slag contents experienced a decline in strength at all ages. However, the greater the amount of SS incorporated, the lesser the degree of drying shrinkage. The compressive strength of all groups was improved, and the drying shrinkage was reduced by carbonation treatment. The samples with 5%, 10%, and 15% SS content exhibited shrinkage rates of 2.19%, 1.74%, and 1.60%, respectively, after 28 days of curing. The reason was that after carbonation treatment, hydrated magnesium carbonates (HMCs) were generated in the SS–MSH cement, and a Ca–Mg–C amorphous substance formed by hydration and carbonation of C_2_S in steel slag filled in the pores, which enhanced the density of the matrix, improved the compressive strength of the specimen, and reduced the shrinkage rate.

## 1. Introduction

At present, magnesium cementitious materials primarily consist of magnesium oxysulfate (MOS) cement [1,2], magnesium oxychloride cement (MOC) [3], magnesium phosphate cement (MPC) [4], and magnesium silicate hydrate (MSH) cement [5]. The main component of these types of magnesium cement is magnesia powder. Compared to Portland cement and calcium sulfoaluminate cement, magnesium cement exhibits advantages such as higher strength, superior adhesion, reduced CO_2_ emissions, and environmental sustainability [6,7,8,9,10]. Among these, MSH cement has recently emerged as a novel type of cement, which is composed of silica fume (SF) and light burned magnesia (LBM). Upon contact with water, it forms an M–S–H gel, offering benefits such as low energy consumption, minimal environmental pollution, carbon negativity, and reduced alkalinity [11,12]. Furthermore, MSH cement can effectively address the environmental issues caused by calcium cement and can make practical use of low-grade magnesite resources that are otherwise unsuitable for chemical applications, aligning with sustainable development. Consequently, they hold significant promise in the field of construction [13].

Steel slag (SS) is a byproduct of the steel-making process. China discharges over 100 million tons of SS annually, but only approximately 10% is effectively utilized [14,15]. The accumulation and disposal of this vast amount of SS poses environmental risks [16]. Chemically, SS contains a considerable amount of calcium oxide, and its primary mineral phases include dicalcium silicate (C_2_S), tricalcium silicate (C_3_S), and tetracalcium aluminoferrite (C_4_AF)—all of which exhibit a certain degree of reactivity [17,18]. However, the high concentrations of CaO and MgO in SS compromise its stability [19]. Recent studies indicate that the stability of SS can be enhanced through carbonation [20]. Due to the high content of belite (C_2_S) and lime (CaO), SS demonstrates excellent carbonation reactivity, as illustrated in Equations (1) and (2) [21].

2CaO × SiO_2_ + (2 − x) CO_2_ + nH_2_O → xCaO × SiO_2_ × nH_2_O + (2 − x) CaCO_3_ (1)

Ca(OH)_2_ + CO_2_ → CaCO_3_ + H_2_O (2)

Numerous scholars have researched the carbonation treatment of SS. For instance, Ghouleh et al. [22] found that the mechanical strength of SS could reach up to 78 MPa after undergoing accelerated carbonation treatment. Further research by Ghouleh et al. [23] demonstrated that when SS was carbonated under a pressure of 0.15 atmospheres for two hours, the strength of its products was improved, and a certain proportion of carbon dioxide could also be fixed. In a study conducted by Mo et al. [24], carbonation curing was applied to SS paste and concrete samples under specific conditions. As a result, significant improvements were observed in the volume stability of the concrete. Additionally, research by Bodor et al. [25] suggests that the ease of carbonation for various products in SS varies, and different carbonation conditions can result in the formation of distinct carbonated products.

Indeed, there is a growing body of research focusing on the carbonation of MgO cement. After carbonation, various compounds, such as lansfordite (MgCO_3_·5H_2_O), artinite (MgCO_3_·Mg(OH)_2_·3H_2_O), nesquehonite (MgCO_3_·3H_2_O), and hydromagnesite (4MgCO_3_·Mg(OH)_2_·4H_2_O), are formed [26]. Kuenzel et al. [27] studied hydromagnesite and found that it can react with MgO or Mg(OH)_2_ to form a hydraulic basic magnesium carbonate and even a novel type of magnesium carbonate cement. Hu et al. [20] investigated the impact of SS hydration and carbonation on the properties of MOS cement. By incorporating varying proportions of SS, the study explored the effects of different carbon dioxide pressures on the modified MOS cement, ultimately identifying the most effective carbonation parameters and the cement products with the highest compressive strength. Ba et al. [28] discovered that carbon dioxide carbonation can improve the pore structure of MOS cement, thereby enhancing its compressive strength. Finally, Li et al. [29] achieved improved water resistance in cement products through carbonation modification using ground granulated blast-furnace slag added to MOS cement.

Although research has been conducted on the effects of carbonation on the properties of SS–magnesium cementitious materials, studies on the carbonation performance of MSH cement are comparatively scarce. The primary hydration product of MSH cement is the gel phase, which contains a significant amount of structural water. Inadequate curing conditions can result in the loss of crystalline water, causing shrinkage in MSH cement [30]. Consequently, it is crucial to control the shrinkage rate of MSH cement to a minimal extent during its actual service life. To address this issue, the present study improves the excessive drying shrinkage of MSH cement using the hydration and carbonation of SS.

The objective of this study is to investigate the impact of carbonation on the properties of MSH cement incorporating SS. This paper systematically analyses the influence of SS dosage, carbon dioxide partial pressure, and carbonation curing time on the compressive strength, shrinkage rate, phase composition and content, as well as the microstructure of MSH cement. By deeply exploring the effects of carbonation on SS–MSH cement, we aim to identify the optimal SS dosage to improve the shrinkage characteristics of MSH cement, thereby enhancing its practical application in construction engineering.

## 2. Materials and Methods

### 2.1. Raw Materials

**Figure 1:**
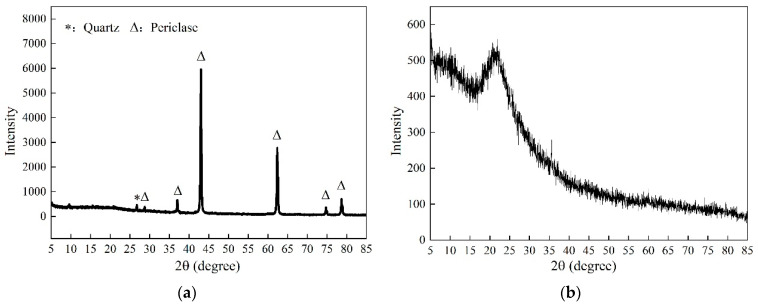
XRD patterns of the main raw materials of MSH cement: (**a**) MgO; (**b**) SF.

The primary raw material used in these experiments was high-reactivity magnesia powder (LBM), sourced from Haicheng City in Liaoning Province. LBM is generally formed by calcining magnesite (mainly composed of MgCO_3_) at 700–900 °C. It contains more than 80% magnesia. The chemical composition is tabulated in Table 1. X-ray diffraction (XRD) analysis of the raw materials used in this study reveals that the peak positions are primarily MgO, as shown in Figure 1a. SF was purchased from Elkem China, with the model being 95U; its phase analysis is shown in Figure 1b. SS came from the Ansteel Group, and it is a basic oxygen furnace SS. SS was ground using a vibratory ball mill. It was initially hammered into pieces smaller than 50 mm, then crushed to below 5 mm using a jaw crusher, and finally ground using a vibratory mill. Each grinding session involved approximately 100 g of material. Additionally, the active MgO content in the LBM, as determined by the hydration method, was 72.5%.

**Figure 2:**
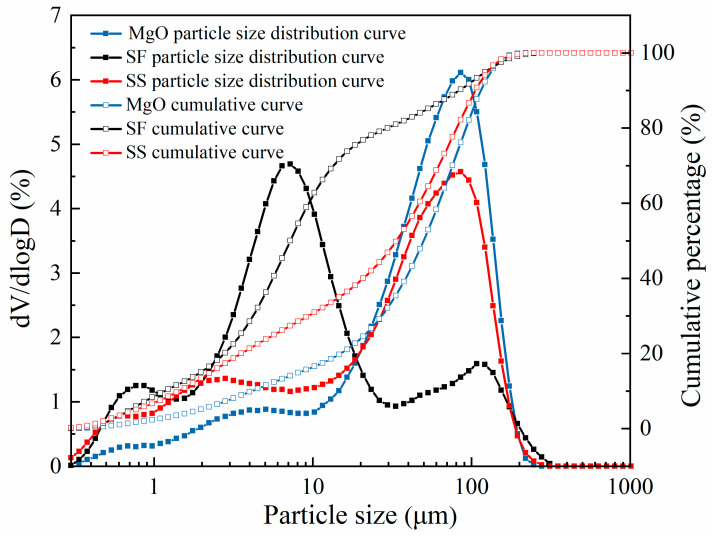
Particle size distribution of MgO, SF, and SS (particle size distribution curve and cumulative curve).

Particle size analysis of MgO, SF, and SS was conducted using a laser particle size distribution analyzer, as depicted in Figure 2.

### 2.2. Experimental Methods

Zhang et al. [12] found that the inclusion of sodium hexametaphosphate can elevate the alkalinity of cement in the early stages of hydration, thereby facilitating the progress of the hydration reaction. Furthermore, it was discovered that the highest amount of M–S–H gel was generated when the Mg/Si ratio ranged between 2/3 and 1. Accordingly, this study employed a Mg/Si ratio of 2/3, a water/cement ratio of 1/2, and a sodium hexametaphosphate content of 2% to produce MSH cement [31].

The mix proportions for this study are presented in Table 2. High-reactivity LBM and SF were weighed according to a Mg/Si ratio of 2/3, a sodium hexametaphosphate content of 2%, and a water/cement ratio of 1/2. In compliance with the JC/T 729–2005 standard [32], sodium hexametaphosphate was first dissolved in water. Subsequently, SS, LBM, and SF were mixed in a mixer to prepare the MSH cement paste. Initially, the mixture was stirred at a low speed of 60 r/min for 120 s. After a 15 s pause, high-speed stirring was performed at 300 r/min for another 120 s. The SS–MSH cement paste was then poured into polypropylene molds with dimensions of 40 × 40 × 160 mm. Samples were cured in a humidity chamber with a relative humidity of 60 ± 5% and a temperature of 25 ± 2 °C before demolding.

For air-cured samples, the molds were removed 24 h after pouring, followed by curing in a chamber with 60 ± 5% humidity and 25 ± 2 °C temperature for 28 days. For CO_2_-cured samples, two sets were prepared under normal pressure (1 atm) and high pressure (5 atm). The normal pressure group was demolded 24 h after pouring and placed in a carbonation chamber with a CO_2_ pressure of 1 atm for 4 h. Thereafter, they were moved back to the curing chamber for 28 days. The high-pressure group was transferred to the curing chamber for 6 days, followed by pressurized carbonation (5 atm) for one day and subsequently returned to the curing chamber for further curing until the total curing time reached 28 days. This was due to the high reactivity of MgO in the early stage of hydration, and the reaction of high-pressure carbonation was violent. Therefore, the specimen was cured for 6 days before carbonization.

### 2.3. Test and Analysis Methods

Compressive strength tests on MSH cement blocks were conducted using a fully automatic constant stress testing machine (DYE-300S, Cangzhou Jingwei Instrument Equipment Manufacturing Co., Ltd., Cangzhou, China). The smooth, flat sides of the samples were placed at the center of the testing machine and loaded at a rate of 1 kN/s, with the device’s maximum load capacity being 300 kN.

The shrinkage rate of the samples was measured according to the construction materials industry standard JC/T 603–2004 [33]. A length comparator (ISOBY-160, Diao Jianyi company, Changji, China) was used to determine the length of the samples. Subsequently, the samples were removed, and the length variations were measured at 3, 7, 11, 14, 17, 21, 24 and 28 days post-pouring. The shrinkage rate of the cement paste samples was calculated using Equation (3):

(3) $$\varepsilon_{st}=\frac{l_{0}-l_{1}}{160}\times 100\%$$

where *ɛ_st_* represents the shrinkage rate (%), *l*_0_ denotes the initial length of the samples (mm), *l*_1_ symbolizes the length of samples at different ages (mm), and 160 indicates the effective length of the specimen (mm).

XRD analyses were performed on the dried powder samples using a PANalytical X’Pert powder X-ray diffractometer (Malvern Instruments Limited and PANalytical B.V. Malvern, UK). The device utilized a copper target, operated at a voltage of 40 kV, and a current of 40 mA. The scan range was set between 5.0° and 85.0° (2θ), with a step size of 0.02°.

For microscopic morphology observations, the required test blocks were crushed into flat, thin pieces measuring between 3–5 mm and analyzed using a scanning electron microscope (SEM) (Zeiss Sigma HD, Jena, Germany). Energy-dispersive X-ray spectroscopy (EDS) was employed to analyze the elemental composition and ratios.

Approximately 1.120 g of small test blocks that met the test requirements were subjected to pore size distribution tests using a mercury intrusion porosimeter (MIP; AutoPore IV 9500, Boynton Beach, FL, USA) at a curing age of 28 days. The pore structure composition was analyzed based on the test results. A comprehensive thermal analyzer (STA 449F3, Netzsch, Selb, Germany) was used for thermogravimetric (TG) and differential scanning calorimetry (DSC) analyses. A nitrogen atmosphere was employed, and the temperature was increased at a rate of 10 °C/min up to 1000 °C to observe all changes during the heating process. Finally, 1.0 g of sample powder was placed in 10 mL of deionized water. Following 20 min of ultrasonic vibration, the solution was allowed to stand for 12 h. The supernatant was collected, and the pH was gauged using a pH meter (model: PHS-3C, INESA Scientific Instrument, Shanghai, China) to analyze pH variations. The measurement of setting time was performed using vicat apparatus according to Chinese standard GB/T 1346–2001 [34].

## 3. Results and Discussion

### 3.1. Effect of Atmospheric Carbonation on SS–MSH Cement

#### 3.1.1. Setting Time

**Figure 3:**
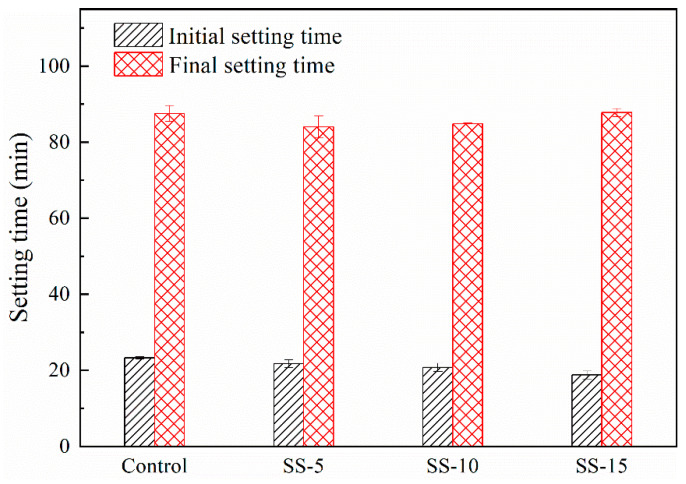
Setting times of MSH cement mixed with varying proportions of SS.

Figure 3 presents the initial and final setting times of MSH cement mixed with varying proportions of SS. It can be observed that the initial and final setting times of MSH cement decrease slightly upon SS addition. Specifically, when 15% SS was added, the initial setting time was reduced from 23.5 min to 19.5 min, while the final setting time essentially remained at 87.5 min.

#### 3.1.2. Compressive Strength

**Figure 4:**
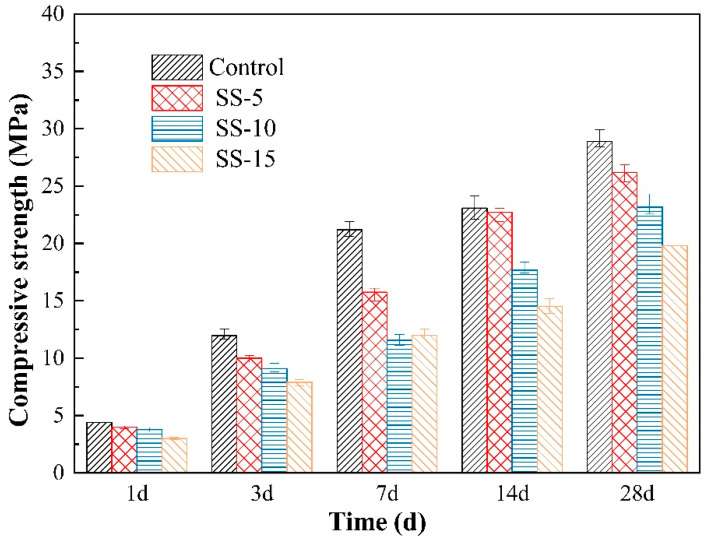
Compressive strengths of MSH cement mixed with varying proportions of SS under atmospheric conditions.

Figure 4 depicts the relationship between the compressive strength of non-carbonated samples and the SS dosage. This reveals that the compressive strength of MSH cement significantly declines upon SS incorporation. At 1 day of curing, the strengths of MSH cement with 0–15% SS were measured at 4.4 MPa, 4 MPa, 3.8 MPa, and 3 MPa, respectively. After 28 days of curing, the compressive strengths dropped from 28.9 MPa to 26.2 MPa, 23.2 MPa and 19.8 MPa, respectively. This suggests that adding SS hinders the strength development of MSH cement, which is correlated with the high pH of SS. A higher pH environment is unfavorable for the growth of M–S–H gel in MSH cement, leading to a decrease in the sample strength [35]. The pH of SS is approximately 13, whereas the pH of MSH cement is approximately 10. Thus, under atmospheric conditions, the SS inclusion adversely impacts MSH cement.

**Figure 5:**
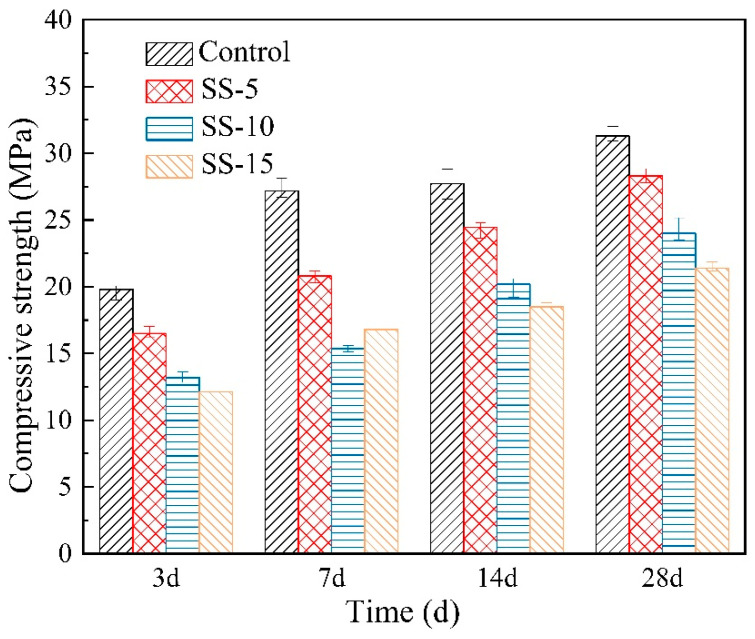
Compressive strengths of MSH cement after atmospheric carbonation curing.

Figure 5 presents the compressive strength of each test block after atmospheric carbonation. Interestingly, carbonation positively affects the compressive strength of MSH cement mixed with SS. After three days of curing, the compressive strengths of the control group and the samples with 5, 10 and 15 wt.% SS increased by 65.2%, 65%, 45%, and 53%, respectively, compared to the non-carbonated states. After 28 days, the compressive strengths elevated by 108%, 107%, 103%, and 109%, respectively, indicating that carbonation enhances the strength of SS–MSH cement. Furthermore, the carbonation efficacy does not deteriorate with an increasing SS amount, thus suggesting that the strength of the post-carbonated SS–MSH system is improved.

#### 3.1.3. Shrinkage Rate

**Figure 6:**
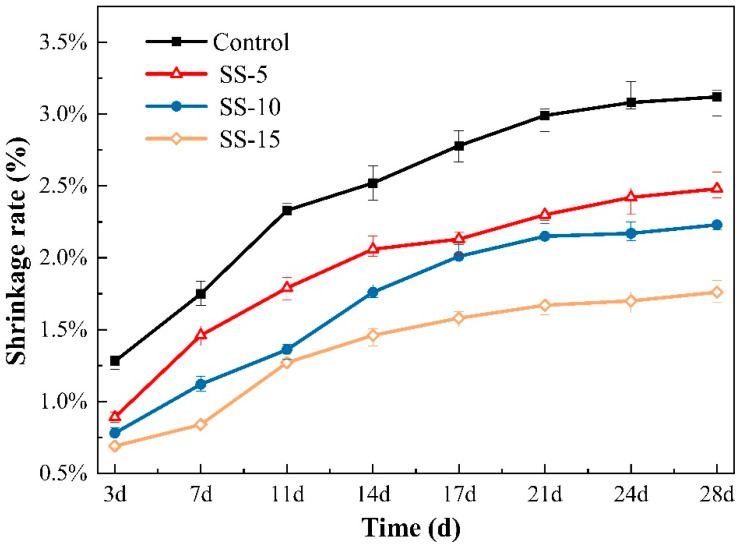
Shrinkage rates of MSH cement mixed with varying proportions of SS under atmospheric conditions.

Figure 6 displays the changes in the shrinkage rate for different sample groups under atmospheric conditions at various ages. According to the data presented in Figure 6, the 28-day shrinkage rate of MSH cement paste stood at 3.12%. This is primarily related to the loss of bound water in the system. Due to the preponderance of gel-like substances within pure MSH, which are less stable than crystals, severe shrinkage occurs in MSH cement when the external moisture level falls below the internal moisture level. At this point, bound water and some of the crystalline water within the system are released.

On the third day, the shrinkage rates for the control group and the cement samples with 5%–15% SS addition were 1.28%, 0.89%, 0.78%, and 0.69%, respectively. These numbers indicate that higher SS levels result in lower levels of shrinkage, albeit at the cost of reduced strength. When the added SS amount increased from 0% to 5%, the shrinkage rate decreased by 0.39%. A smaller reduction of only 0.11% was observed when the SS content was increased from 5% to 10%. Even though SS contains RO phases and the hydration products formed by substances such as C_2_S have good crystallinity, an excessive pH is not conducive to the formation of M–S–H gels. Consequently, the hydration level is low, leading to a noticeable decline in strength and the manifestation of shrinkage.

When naturally cured for up to 28 days, all sample groups exhibit significant shrinkage, which decreases with increasing SS content. Specifically, as the SS content increased from 5% to 15%, the 28-day shrinkage rate decreased from 2.48% to 1.76%. Importantly, it can be discerned from Figure 6 that the main changes in MSH cement shrinkage occur within the first 14 days of hydration, and the system tends to stabilize thereafter.

**Figure 7:**
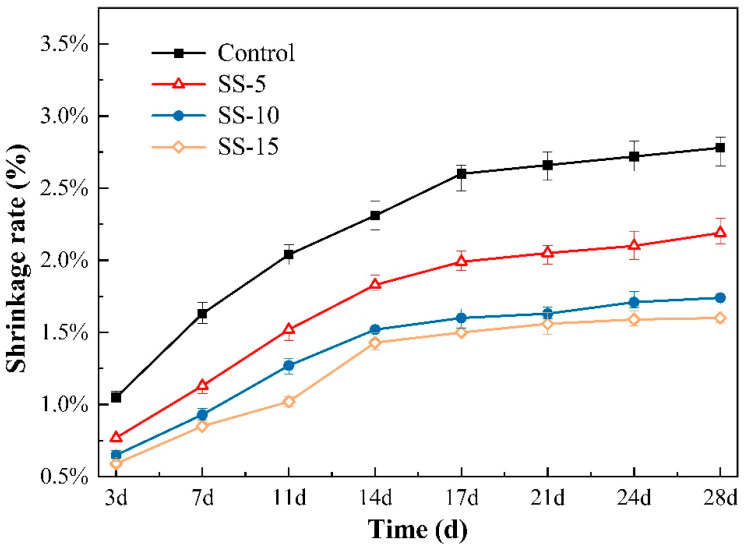
Shrinkage rates of MSH cement mixed with varying proportions of SS under carbonation curing.

Figure 7 illustrates the shrinkage rates for various sample groups after undergoing carbonation curing. Remarkably, the shrinkage rates of all the groups decreased when compared to those undergoing atmospheric conditions. This change is chiefly associated with the reaction between carbon dioxide and CaO and MgO, resulting in the formation of calcium and magnesium carbonates. These carbonates are stable and well dispersed within the matrix, mitigating volume shrinkage.

As depicted in Figure 7, the shrinkage rates after three days of curing were 1.05%, 0.77%, 0.65%, and 0.59%, respectively. When extended to 28 days of curing, the rates were 2.78%, 2.19%, 1.74%, and 1.60%, respectively. Thus, carbonation significantly decreases the extent of shrinkage. The principal shrinkage occurs within 14 days, pointing to the major hydration reactions taking place during this period. After 14 days, unreacted MgO participates in hydration, increasing the volume and compensating for shrinkage, although this slight expansion is relatively minimal when compared to the overall volume shrinkage.

#### 3.1.4. XRD Analysis

**Figure 8:**
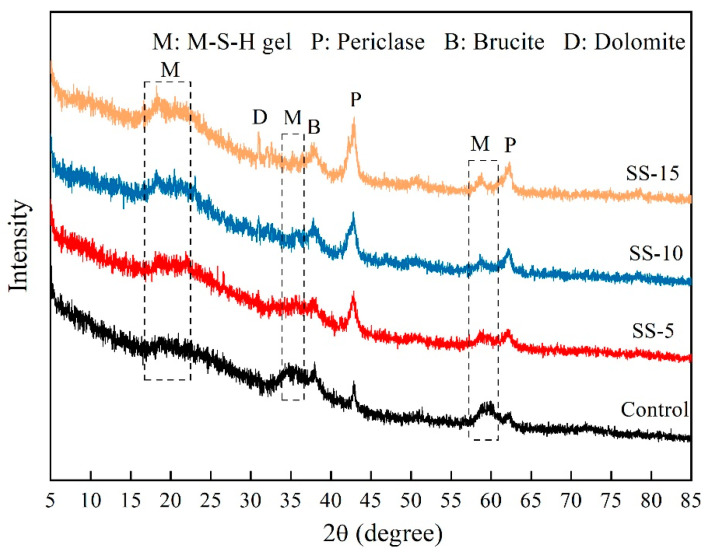
XRD patterns of MSH cement mixed with varying proportions of SS after 28 days under atmospheric conditions.

Figure 8 provides the diffraction patterns of MSH cement blocks mixed with varying proportions of SS after 28 days of atmospheric conditions curing. Distinct dispersion peaks of M–S–H gel in the SS-free samples can be observed at approximately 19.8°, 35°, and 59.5° after 28 days of curing. After the addition of SS, the peak intensity of MgO increases with the rising SS proportion. Concurrently, the dispersion peak of the M–S–H gel phase weakens, while the phase peak of MgO intensifies. At 15% SS addition, Ca^2+^ and Mg^2+^ ions react with ambient CO_2_ to form dolomite. Furthermore, the peaks for Mg(OH)_2_ strengthen, signifying increased formation of Mg(OH)_2_ and resulting in a decline in the test block strength.

**Figure 9:**
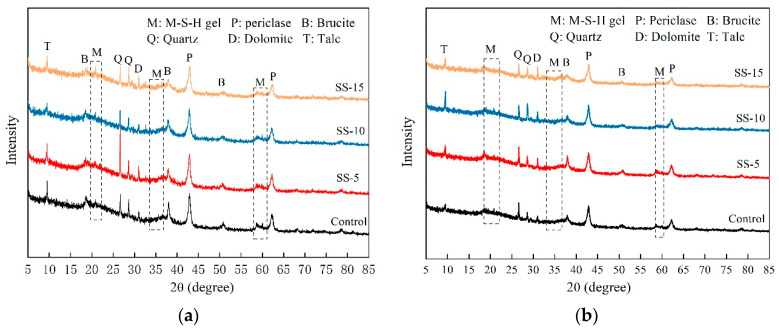
XRD patterns of SS–MSH cement after carbonation for: (**a**) 7 days; (**b**) 28 days.

Figure 9 presents the XRD spectra for MSH cement samples with varying SS additions of 0, 5, 10 and 15 wt.%, after different periods of carbonation curing. From Figure 9a, it is apparent that the primary compounds after seven days of carbonation curing include talc, Mg(OH)_2_, dolomite, and unreacted MgO and SF. Compared to naturally cured samples, the carbonated samples contain a significantly larger amount of dolomite phase. Compared to the MSH cement samples without SS, the phase peak intensity of Mg(OH)_2_ in the samples with SS weakens. This suggests that due to the presence of SS, the pH level increases, which is not conducive to the hydration of MgO, resulting in a slower reaction and impeding the formation of the M–S–H gel. Consequently, this is detrimental to the development of compressive strength. Therefore, as the SS proportion increases, the compressive strength of the samples exhibits a declining trend.

Figure 9b displays the XRD spectra for SS–MSH cement samples after 28 days of curing. As the carbonation time increases, the peaks corresponding to MgO weaken, and those related to Mg(OH)_2_ nearly vanish. Meanwhile, the diffusion peaks for the M–S–H gel develop robustly. This is primarily due to the fact that carbonation lowers the pH of the system, thereby fostering the growth of the M–S–H gel [36,37].

#### 3.1.5. SEM Analysis

**Figure 10:**
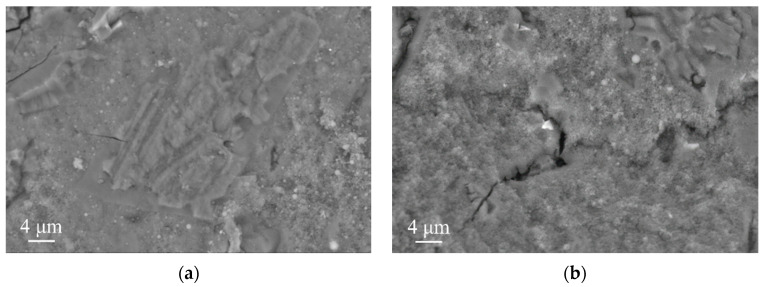
SEM images for 28-day hydrated MSH cement samples: (**a**) control group; (**b**) SS-15.

Figure 10 provides SEM images for 28-day hydrated MSH cement samples. As revealed in Figure 10a, the control group presents a relatively smooth microstructure. Its primary constituents are M–S–H gel, minor amounts of Mg(OH)_2_, and spherical SiO_2_. Figure 10b shows SEM images for 28-day naturally cured MSH cement with 15% SS. It is apparent that adding SS produces numerous cracks, and the structure becomes more porous. Therefore, compared with the control group, the samples with a 15% SS addition showed a decreasing trend in strength, which was consistent with Figure 4. Furthermore, the morphologies of the M–S–H gel remain largely unchanged. An abundance of amorphous Ca–Mg compounds, originating from the SS’s Ca^2+^ content, encapsulates MgO, impeding its complete hydration and resulting in a lower test block strength.

**Figure 11:**
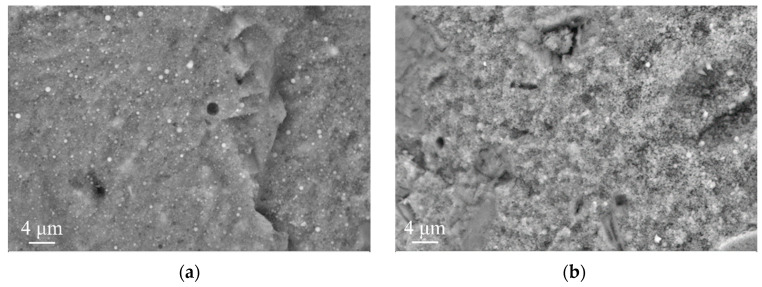
SEM images for MSH cement samples after 28 days of curing: (**a**) control group; (**b**) SS-15.

Figure 11 shows SEM images for MSH cement samples after 28 days of curing. Again, the control group shows a relatively smooth microstructure. The M–S–H gel reacts with CO_2_ to form hydrated magnesium carbonates (HMCs), which fill the pores, thereby enhancing the strength of the carbonated specimens. After undergoing carbonation with 15% SS, the carbonation-cured samples obviously produce more crystal structures. Compared with the uncarbonized sample with 15% SS, these crystals act as a skeleton filler, thereby increasing the strength of the specimen. Since the hydration products of MSH cement are dominated by the gel phase, which is prone to drying and shrinkage, a proper crystal gel ratio is favorable for strength improvement. The introduction of SS and carbonization improves the crystal gel ratio of the MSH cement and thus increases the strength of the specimen blocks. Amorphous substances in the post-carbonation samples are transformed into amorphous Ca–Mg–C compounds, improving the compressive strength of MSH cement and its resistance to shrinkage, corroborating the results of linear expansion tests.

### 3.2. Effect of High-Pressure Carbonation on SS–MSH Cement

#### 3.2.1. Compressive Strength

Figure 4 illustrates that prior to carbonation, the compressive strength of the samples diminishes as the amount of SS admixture increases. In the uncured 7-day samples, the low compressive strength primarily emanates from the formation of Mg(OH)_2_ during the initial seven days of the hydration reaction. Characterized by its porous nature and low strength, Mg(OH)_2_ gradually diminishes as the denser M–S–H gel forms with the continuation of the hydration process.

**Figure 12:**
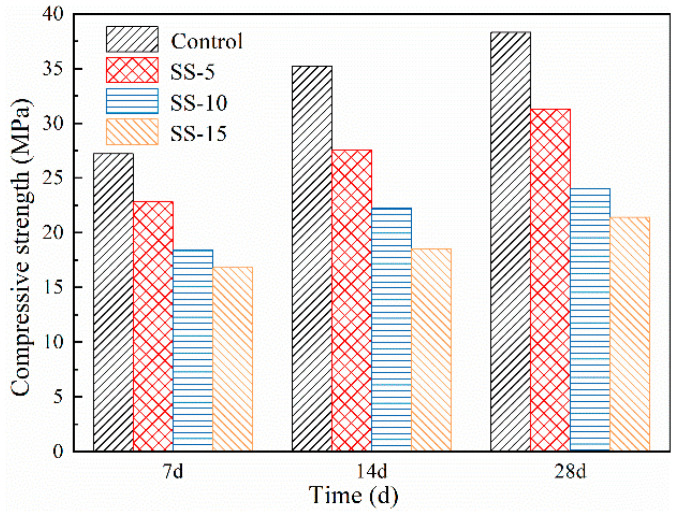
Compressive strengths of MSH cement mixed with varying proportions of SS under high pressure carbonation.

From Figure 12, it is discerned that post-carbonation, the compressive strength of the samples improves. This can be attributed to two factors: first, the reaction between Mg(OH)_2_ and CO_2_ results in a significant amount of HMCs; second, carbonation is an exothermic reaction, the heat from which potentially accelerates the hydration of MSH cement. Remarkably, the 7-day cured samples demonstrate a prominent increase in strength post-carbonation, with samples containing 0–15% SS exhibiting strengths that are 128%, 145%, 158%, and 140% of their naturally cured counterparts.

Carbonation leads to the hydration of C_2_S present in SS, forming a C–S–H gel. Thus, the rate of compressive strength increase in the post-carbonation MSH cement samples correlates positively with the SS content. However, the improvement in the compressive strength becomes less noticeable after 28 days of curing. For instance, MSH cement samples with 0–15% SS admixture experienced an increase in 28-day compressive strength from 28.9, 26.2, 23.2 and 19.8 MPa to 38.3, 31.3, 24 and 21.4 MPa, respectively. This indicates that the MSH cement with SS underperforms in terms of carbonation-induced performance enhancements compared to the conventional MSH cement.

#### 3.2.2. Mass Loss

**Figure 13:**
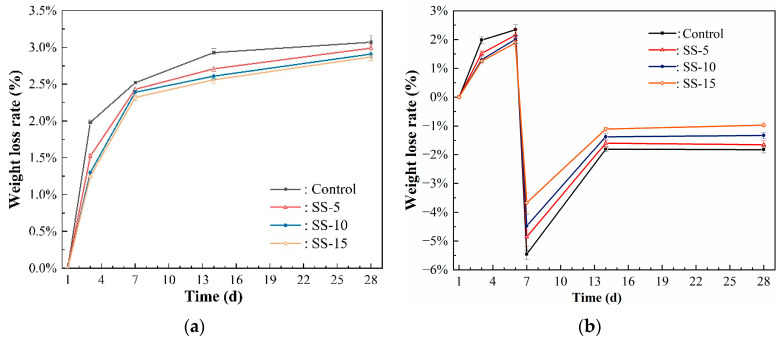
Mass loss diagrams of MSH cement mixed with varying proportions of SS: (**a**) atmospheric conditions group; (**b**) carbonization group.

Figure 13 shows the mass loss in SS–MSH cement samples under different curing periods. As evident from Figure 13a, before carbonation, the primary mass loss in the samples mainly occurs before the 14-day aging period. This loss is principally attributed to the evaporation of free water and crystalline water within the MSH cement. Post-14-day curing, this loss reaches its maximum value and consequently levels off, and the hydration reaction of MSH cement progresses towards completion. Upon undergoing pressurized carbonation, all samples exhibit a net increase in mass between 6 and 7 days due to the formation of a large amount of HMCs. However, between 7 and 14 days post-carbonation, a certain loss of mass of those samples was observed compared to the 7th-day after carbonization, which was attributed to inadequate humidity in the curing chamber to compensate for the internal loss of free water, resulting in shrinkage. Beyond the 14-day mark, mass loss is negligible for both the atmospheric conditions and pressurized carbonation groups (5 atm). Consequently, it can be concluded that the hydration reaction in the MSH cement system primarily occurs within the first 14 days.

#### 3.2.3. Shrinkage Rate

**Figure 14:**
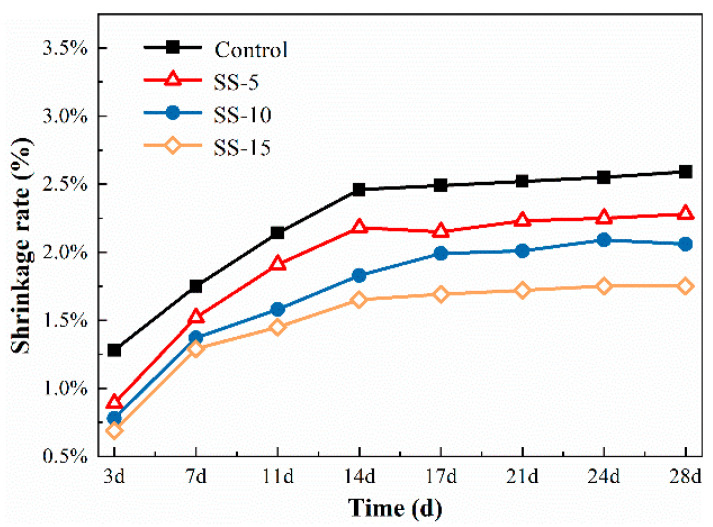
Drying shrinkage of MSH cement mixed with varying proportions of SS under high pressure carbonation.

Figure 6 and Figure 14 delineate the changes in the shrinkage rate of SS–MSH cement before and after high-pressure carbonation. It is evident that the shrinkage rate for the control group reached 3.12% after 28 days of curing without carbonation. In contrast, the introduction of SS has a mitigating effect on the shrinkage rate, owing to the expansive action of free CaO present in the SS, which decreases the expansion coefficient of the SS–MSH cement samples. Furthermore, the addition of SS results in the formation of C–S–H gel due to the reaction between Ca^2+^ and SiO_2_, thereby inhibiting shrinkage. After undergoing pressurized carbonation, the samples with 5–15% SS content exhibited shrinkage rates of 2.28%, 2.06%, and 1.75% after 28 days of curing. This suggests that carbonation also contributes to the reduction in the shrinkage rate in MSH cement. The underlying reason is that carbonation leads to the formation of HMCs, filling the voids in the M–S–H gel structure and thus decreasing the shrinkage rate.

#### 3.2.4. pH Change

**Figure 15:**
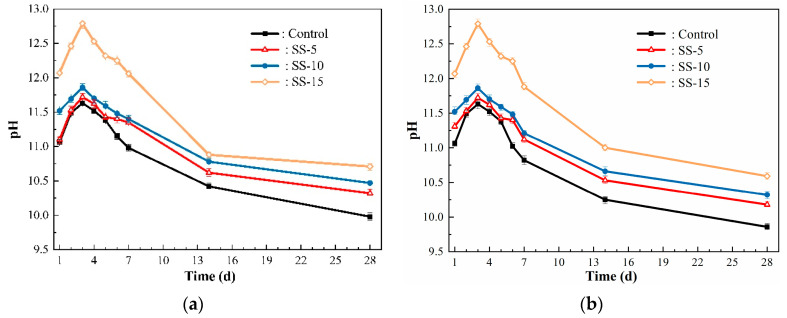
pH of MSH cement mixed with varying proportions of SS: (**a**) atmospheric conditions group; (**b**) pressurized carbonation group.

Figure 15 represents the pH changes in MSH cement samples with 0–15 wt.% SS content over different curing periods. From Figure 15a, it is observed that the overall pH environment of the SS–MSH system markedly rises as the SS content increases. Specifically, the general trend shows a pH increase during the initial one to three days, followed by a gradual decline thereafter. Both the carbonated and naturally cured groups reached their peak pH on the third day, with the samples containing 15% SS reaching a pH of 12.79 after three days of hydration. After undergoing pressurized carbonation on the 6th day, the 7th-day pH level significantly dropped compared to the normal temperature cured group. By the time the samples reached the 14th day of curing, the pH stabilized. At the 28-day mark, the pH levels for the naturally cured samples containing 0–15% SS were 9.98, 10.32, 10.47, and 10.71, respectively. Meanwhile, the post-carbonation pH levels decreased to 9.86, 10.18, 10.32, and 10.59, respectively. This implies that carbonation effectively lowers the internal pH of the SS–MSH cement system, thereby promoting the growth and development of the M–S–H gel, which in turn enhances the compressive strength of the SS–MSH cement samples [11].

#### 3.2.5. TG–DSC Analysis

**Figure 16:**
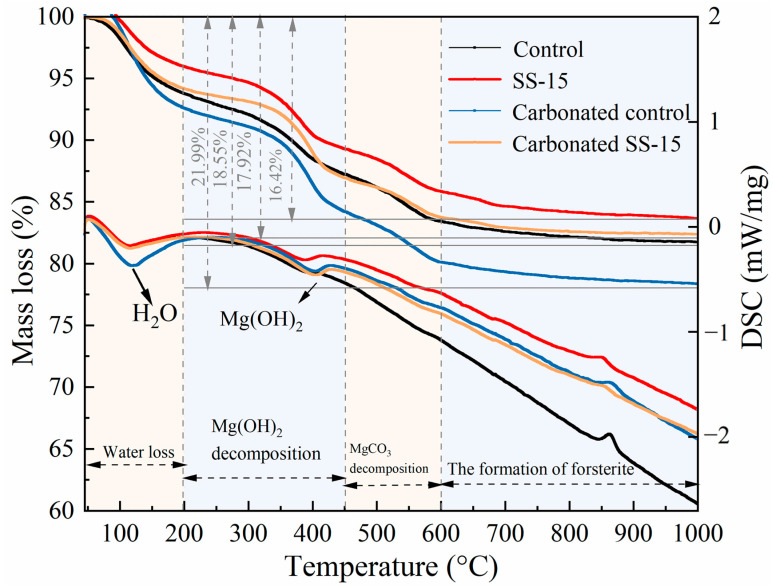
TG–DSC curves of SS–MSH cement at 28 days curing.

Figure 16 illustrates the TG–DSC results of the samples before and after carbonation. Prior research [38,39,40], indicates that the thermal decomposition process of MSH cement can be divided into four stages. The first stage occurs between 50 and 200 °C, during which the mass loss is attributed to the evaporation of free and bound water from the reaction products. The second stage spans 200–450 °C and is characterized by the decomposition of Mg(OH)_2_ into MgO and H_2_O. The third stage, ranging from 450 to 600 °C, involves the decomposition of HMCs formed through carbonation. Last, the fourth stage occurs above 600 °C, where forsterite is generated.

The TG–DSC results displayed in Figure 16 fully corroborate the above descriptions. The mass losses for the control group, SS-15 group, carbonated control group, and carbonated SS-15 group were 18.55%, 16.42%, 21.99%, and 17.92%, respectively. Subsequent to carbonation, the DSC curve reveals four distinct exothermic and endothermic valleys, corresponding to four episodes of mass loss in the TG curve. These valleys occur at approximately 100 °C, 400 °C, 550 °C, and 850 °C, signifying dehydration, dehydroxylation of Mg(OH)_2_, decomposition of HMCs into MgO and CO_2_, and the formation of forsterite, respectively.

#### 3.2.6. Porosity

Table 3 presents the MIP results for SS–MSH cement samples cured for seven days. The porosity of the cement directly affects its compressive strength. Generally, higher porosity correlates with lower strength. Table 3 shows that the inclusion of SS in the normal temperature cured samples results in a noticeable increase in total porosity, particularly in pores greater than 100 nm in diameter. This can be attributed to the high alkalinity of SS disrupting the M–S–H gel structure, while its free calcium oxide induces an expansion that enlarges the pores within the samples. After undergoing pressurized carbonation, the porosity in the carbonated samples diminishes, suggesting that a series of carbonates have filled the voids, enhancing the compressive strength of the samples. However, due to the test’s 7-day period, the proportion of gel pores smaller than 10 nm is relatively low. This not only indicates limited early-stage M–S–H gel formation but also explains why the early-stage compressive strength of MSH cement is relatively low.

**Figure 17:**
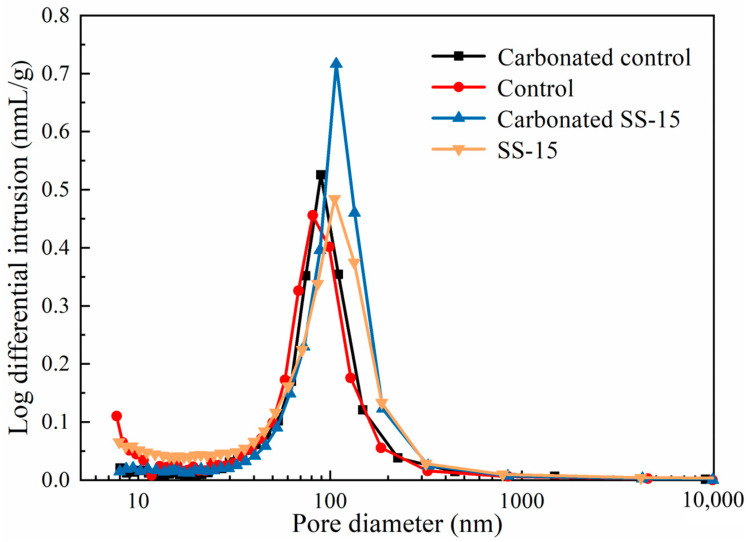
Pore size distribution of MSH cement.

Figure 17 delineates the pore size distribution in the control and SS-15 samples that have undergone pressurized carbonation and natural curing. Post-carbonation, the control samples exhibited a decrease in micropores but an emergence of mesopores with diameters greater than 100 nm. This is attributable to a certain expansion caused by the formation of carbonation products, leading to an enlargement of pores from the 10–100 nm range to above 100 nm. Incorporating 15% SS results in a higher total porosity compared to the control samples. However, the porosity of SS-15 samples decreases upon carbonation, making the matrix denser and leading to the formation of capillaries smaller than 10 nm. Consequently, an increase in compressive strength is observed.

## 4. Conclusions

This study has undertaken an exhaustive analysis and research on the properties of MSH cement mixed with varying proportions of SS under both atmospheric conditions and carbonated curing conditions. Focal areas include mechanical strength and shrinkage rate studies, exploring pH changes pre- and post-carbonation as well as in the early stages of hydration. Advanced techniques such as XRD, SEM, TG, and MIP were employed to investigate the mineral composition and microstructure of SS–MSH cement. Based on this research, the following conclusions can be drawn:

- Under atmospheric conditions, MSH cement mixed with different SS contents experiences a decline in strength at all ages, decreasing progressively with increasing SS content—from 28.9 MPa down to 19.8 MPa. The greater the SS amount incorporated into MSH cement, the less shrinkage is observed at various aging periods. After carbonation treatment, the compressive strength increases compared with the uncarbonized samples with the same SS content, and shrinkage rates also decrease, with the MSH cement containing 15 wt.% SS showing the most significant improvements.
- Upon the addition of SS, the presence of free calcium oxide and alkali metal ions in the SS elevates the pH of the MSH cement system. Furthermore, the pH increases in correlation with the amount of SS added. As the pH rises, the solubility of SF decreases, affecting the formation of the M–S–H gel and consequently leading to a reduction in the compressive strength. As the hydration reaction progresses, the pH of MSH cement reaches its peak at 3 days and subsequently decreases as hydration continues. After undergoing pressurized carbonation treatment, the pH of the SS–MSH cement decreases. For example, the pH value of MSH cement with a 15 wt.% SS addition decreases from 10.71 to 10.59 at 28 days, which is beneficial for gel growth. This is one of the reasons for the observed improvement in the compressive strength following carbonation.
- Microscopic characterization methods such as XRD, TG–DSC, and SEM were employed to analyze the products before and after carbonation. XRD results reveal that the primary hydration products in SS–MSH cement are M–S–H gel, small amounts of Mg(OH)_2_, and some unreacted MgO and SiO_2_. Following carbonation treatment, HMCs are formed within the SS–MSH system. Simultaneously, the C_2_S in the SS undergoes hydration and carbonation to form amorphous Ca–Mg–C substances that fill the pores, thereby enhancing the matrix density. Consequently, the compressive strength of the specimens improves while the shrinkage rate decreases.

In summary, carbonation curing of SS–MSH cement can effectively control the shrinkage rate observed in traditional MSH cement and yield higher compressive strengths.

## Figures and Tables

**Table 1:** Chemical compositions of raw materials (%).

Component	Content (wt.%)					
	MgO	SiO_2_	CaO	Al_2_O_3_	Fe_2_O_3_	Others
LBM	87.63	2.34	2.91	0.42	0.47	6.23
SF	1.19	95.73	0.68	0.22	0.25	1.93
SS	9.19	18.20	37.85	3.66	25	6.10

**Table 2:** Mix proportions of MSH cement.

Samples	Mixture Design (wt.%)			
	SS	MgO	Water	SF
Control	0	40	50	60
SS-5	5	40	52.5	60
SS-10	10	40	55	60
SS-15	15	40	57.5	60

**Table 3:** MIP results of SS–MSH cement samples.

Samples	Total Intrusion Volume	Total Porosity Porosity	Volume of Pores in Each Range (%)		
	(mL/g)	(%)	>100 nm	10–100 nm	<10 nm
Control	0.2122	21.2238	34.13	62.42	3.45
Carbonated control	0.2092	20.9186	54.07	44.83	1.10
SS-15	0.2717	27.1681	53.17	46.07	0.96
Carbonated SS-15	0.2658	26.5781	47.03	49.91	3.06

## Data Availability

Data is unavailable due to privacy or ethical restrictions.
